# The Pesticide Risk Beliefs Inventory: A Quantitative Instrument for the Assessment of Beliefs about Pesticide Risks

**DOI:** 10.3390/ijerph8061923

**Published:** 2011-06-01

**Authors:** Catherine E. LePrevost, Margaret R. Blanchard, W. Gregory Cope

**Affiliations:** 1 Department of Environmental and Molecular Toxicology, North Carolina State University, Box 7633, Raleigh, NC 27695, USA; E-Mail: greg_cope@ncsu.edu (W.G.C.); 2 Department of Science, Technology, Engineering, and Mathematics Education, North Carolina State University, Box 7801, Raleigh, NC 27695, USA; E-Mail: meg_blanchard@ncsu.edu (M.R.B.)

**Keywords:** pesticides, risk communication, pesticide education, science education, inventory

## Abstract

Recent media attention has focused on the risks that agricultural pesticides pose to the environment and human health; thus, these topics provide focal areas for scientists and science educators to enhance public understanding of basic toxicology concepts. This study details the development of a quantitative inventory to gauge pesticide risk beliefs. The goal of the inventory was to characterize misconceptions and knowledge gaps, as well as expert-like beliefs, concerning pesticide risk. This study describes the development and field testing of the *Pesticide Risk Beliefs Inventory* with an important target audience: pesticide educators in a southeastern U.S. state. The 19-item, Likert-type inventory was found to be psychometrically sound with a Cronbach’s alpha of 0.780 and to be a valuable tool in capturing pesticide educators’ beliefs about pesticide risk, assessing beliefs in four key categories. The *Pesticide Risk Beliefs Inventory* could be useful in exploring beliefs about pesticide risks and in guiding efforts to address misconceptions held by a variety of formal and informal science learners, educators, practitioners, the agricultural labor force, and the general public.

## 1. Introduction

Recent discussions of agricultural pesticides in popular media, including the CNN series *Toxic America* [1] and an article in *Newsweek* that relates pesticide exposure to Attention Deficit Hyperactivity Disorder in children [2], are representative of growing national attention to the risks that pesticides may pose to human health. With an increase in public interest, scientists and science educators seek to enhance public understanding of basic toxicology concepts related to pesticides [3]. However, little is known about the current beliefs of the public or of pesticide educators regarding pesticide hazards. Prior to developing communications and curricular materials focusing on pesticide risks for the general public or agricultural audiences, the current knowledge and beliefs of these groups must be characterized. No known quantitative instrument currently captures individuals’ understandings of pesticides and their risks.

In both their working and living environments, pesticide exposure is a significant hazard to farmworkers, who supply the manual labor necessary to cultivate and harvest crops [4]. Therefore, knowledge of basic pesticide toxicology has significant implications for farmworkers’ safety and health, and communication of pesticide risks is essential to preventing pesticide illness and injury. Acute pesticide poisoning has been identified as a public health concern for agricultural workers globally, specifically to those in developing countries [5]. Migrant and seasonal farmworkers are identified as a special risk population because of the cultural and linguistic barriers that these agricultural workers face in maintaining their safety and health within their working environments [4]. In the United States, the majority of hired farm laborers are foreign-born and Spanish-speaking; Mexico is the country of origin for 75% of hired farm laborers, with Central American countries accounting for an additional 2% [6]. Farmworker pesticide educators provide basic pesticide toxicology and safety lessons to farmworkers. Given their critical role in the communication of pesticide risks to the susceptible farmworker population, pesticide educators are an important group for the assessment of beliefs regarding pesticide risk.

This study details the development of the *Pesticide Risk Beliefs Inventory*, including testing with pesticide educators in a southeastern state in the United States, to identify beliefs about pesticides and their risks. A quantitative questionnaire that focuses on pesticide risk beliefs held by scientists, educators, and the general public (ranging from novices to experts) will facilitate the characterization of misconceptions and knowledge gaps, as well as expert-like beliefs. This information will assist curriculum developers in the creation of communications and curricular materials, with the goal of enhancing the audience’s decision-making ability by addressing the most prevalent and pressing discrepancies in expert and audience beliefs.

### 1.1. Theoretical Framework

**Figure 1.**
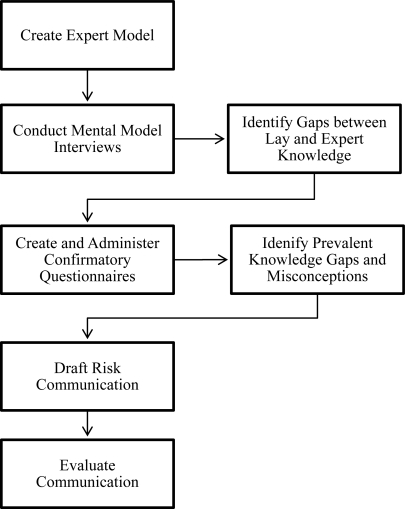
Mental models approach to risk communication, based on Morgan *et al.* [9].

The evolution of risk communication as a field has resulted in greater interest in audience beliefs and in their involvement in the development of communications [7,8]. Morgan *et al.* [9] propose the *mental models* framework, a systematic approach to designing risk communications that emphasizes understanding the knowledge and needs of the target audience in the design process (Figure 1). With its emphasis on capturing how the public conceptualizes risk and its focus on developing the public’s understanding of a hazardous process, the mental models approach reflects this trend of increasing attention to the target audience for risk communications. Mental models are a “set of concepts a person uses to understand and generate inferences about a hazardous process” [10]. Borrowed from cognitive science [11], this approach diverges from previous risk communication frameworks that assumed that an audience knew nothing prior to communication and needed to know nothing more than quantitative estimates related to risk. The risk beliefs comprising individuals’ mental models differ conceptually from hazards (*i.e.*, substances scientifically determined to increase the incidence of an adverse effect through exposure) and risks (*i.e.*, the probability that an adverse effect will result from exposure to a particular substance) [12]. To the extent that beliefs shape practices [13], the emphasis on beliefs in the mental models approach is useful in understanding a target audience’s actions related to a hazard.

Because individuals’ prior knowledge is recognized as being relevant to their decisions regarding risk and their understanding of communications, this framework includes eliciting the audience’s “fragmentary beliefs” about a risk (*i.e.*, mental models) and comparing these beliefs to those of expert models, in order to develop risk messages that address disparities in the models. The terms ‘lay’ and ‘laypeople’ are frequently used to denote the target audience or the general public and are not reflective of the extent of the audience’s knowledge; Fischhoff [14] clarifies that the use of this terminology refers to the *source* of the public’s knowledge (e.g., self-education, indigenous technical knowledge) rather than the *extent* of their knowledge. The ultimate goal of the mental models approach to risk communication is to produce more informed decision-makers.

The mental models approach to risk communication has been used in environmental health communications. For example, homeowners have been the target audience for the design of risk communications pertaining to radon as an environmental contaminant [10,15] and to wildland fires as a natural disturbance and danger to people and the environment [11]. Consumers of pharmaceutical products [16] and of dry cleaning services [17] were the focus for development of mental models for these particular risks. Risk communication related to climate change has employed the mental models approach to understand the general public’s perceptions, misconceptions, and knowledge gaps in this subject [18,19].

Prior to developments in risk communication, workplace safety materials, like material safety data sheets (MSDS), were designed with little attention given to the end users of these safety materials. The mental models approach to risk communication has informed the development of user-friendly materials that address chemical risks in the workplace. Recent research by Kovacs *et al.* [17], Cox *et al.* [20], and Niewöhner *et al.* [21] has resulted in the creation of communications for chemical users in the dry cleaning and electronics industries that are user-centered in content and format and based on user and expert mental models of specific chemicals.

### 1.2. Target Groups

Agricultural audiences do not necessarily believe that pesticides pose a risk to the agricultural community. In a study of Missouri farmers, individuals who own farm land and manage crop production, pesticide exposure was identified as a “concern” by fewer than 50% [22]. One quantitative assessment of farmworkers’ perceived risk was conducted using the Health Belief Model, a framework for public health intervention [23]. Approximately 25% of the 293 North Carolina farmworkers who participated in the study did not perceive pesticides to be a significant risk to themselves, other farmworkers, farmworker children, or unborn children. Study findings from North Carolina suggest that the respondents did not perceive risks to themselves because they do not believe that pesticides can pose risks, rather than because they were using proper techniques and measures to mitigate any such risks. The authors found no relationship between perceived risk and pesticide safety behaviors.

Focus group and interview findings indicate that farmworkers hold many misconceptions about pesticides that can undermine safety measures and increase health risks. For example, several studies found that farmworkers incorrectly believe that pesticide odors can be used to determine when and where pesticides have been applied, as well as the presence of especially toxic pesticides [24,25]. Quandt *et al.* [24] described individuals’ reporting the use of taste, touch, and sight, in addition to smell, as important in detecting pesticides and the potential for pesticide exposure. A qualitative study of farmworkers in North Carolina has shown that participating farmworkers consider natural body openings as the most relevant routes of exposure to pesticides [24]. Inhalation and ingestion were emphasized by farmworkers in the study as points of entry into the body, which is accurate according to expert models. The skin, however, was perceived as an impenetrable barrier to pesticide absorption and as a source of exposure only when pesticides were transferred from the skin to natural openings, when in fact the skin is the largest surface area for pesticide absorption.

Many knowledge gaps in farmworkers’ mental models relate to the long-term health effects of pesticide exposure. Farmworkers were able to identify accurately acute health effects of pesticide exposure (e.g., nausea, vomiting, headaches, dizziness, and skin diseases), yet they were less likely to identify long-term effects, discuss chronic exposure, or describe individuals they knew who had long-term health problems associated with pesticide exposure [24]. Elmore and Arcury [25] reported that few farmworkers believe that adverse health effects resulting from pesticide exposure will last more than one day.

In many cases, these lay beliefs contradict or fail to fully reflect scientific understandings of pesticides, with the potential for increased health risks to farmworkers and their families. Rather than recognizing physical properties as relevant to toxicity, toxicologists typically conceptualize chemicals and their toxicity according to types of pesticides (*i.e.*, fungicides, insecticides, and herbicides) and chemical classes (e.g., organochlorines, organophosphates, and carbamates) [12]. Because the skin is the body’s largest organ, dermal absorption is of great concern among toxicologists, contrary to the lay belief that inhalation and ingestion are the only significant routes of entry. Furthermore, numerous studies have found an association between pesticide exposure and short-term and long-term health effects [4,26]. Acute effects range from mild symptoms, including headaches and dizziness, to more severe effects, such as convulsions and respiratory distress. In the Agricultural Health Study, pesticide applicators in Iowa and North Carolina have been found to have a higher incidence of specific cancers, including prostate cancer, and applicators in North Carolina had higher incidence of multiple myeloma, while overall cancer incidence and mortality rates were lower for applicators than for the general public [27]. This study of pesticide applicators and their spouses suggested the association of pesticide exposure (both globally and related to specific chemicals) with neurological effects like depression and Parkinson’s Disease [28,29], as well as reproductive effects like menstrual cycle influences (e.g., long cycles and missed menstrual periods) [30]. These discrepancies suggest potentially important misconceptions about pesticides that may be widely held among lay audiences.

### 1.3. Research Objective

Specific aspects of lay mental models for pesticide risks have been described in a number of studies, but no single study has examined pesticide beliefs comprehensively. A quantitative inventory for pesticide risk beliefs would facilitate characterization of the beliefs of larger samples and allow for generalizations to be made about prevalent misconceptions and knowledge gaps in pesticide risk beliefs. The objective of this study, therefore, was to develop a pesticide risk belief inventory and test the inventory with one relevant target audience: pesticide educators in a southeastern state in the United States. This is the audience most able to directly impact the pesticide beliefs and knowledge of farmworkers, an at-risk group. As a reminder, ‘lay’ refers to the target audience, pesticide educators, who may or may not have pesticide risk beliefs that match those of experts.

## 2. Methods

Morgan *et al.* [9] formalized their systematic, five-step approach to designing communications in *Risk Communication: A Mental Models Approach*: (1) create an expert model, (2) conduct mental model interviews, (3) create and administer confirmatory questionnaires, (4) draft risk communication, and (5) evaluate communication. Referring to the literature to understand expert mental models and qualitative assessments of lay mental models, this study focused on the third step of the mental models approach. A questionnaire that captured important beliefs from the expert model and misconceptions from the qualitative studies of lay audiences was created and administered.

### 2.1. Inventory Development

Three experts in the field of pesticide toxicology confirmed the expert model of pesticide risks derived from the literature and provided guidance in the selection of facets and items for this inventory. Experts in environmental and molecular toxicology helped to identify the following four facets for measuring pesticide risk beliefs: (1) determination of pesticide risk using physical properties, (2) determination of pesticide risk using chemical properties, (3) association of risk with pesticide routes of entry into the body, and (4) association of risk with adverse health outcomes resulting from pesticide exposure. These facets reflected expert conceptualization of pesticide hazards [4,26], as well as lay beliefs captured in studies of the agricultural community (see Table 1 for inventory facets and items). The inventory was also available in Spanish. The instrument was translated by a third party from English into Spanish and back-translated from Spanish into English to ensure consistency between versions [31].

The inventory contained 19 Likert-type items with six-point scales. A six-point scale was chosen to prevent neutral responses while providing a reasonable range for respondents [32]. Four items corresponded to the facet for determination of risk using physical properties, and three items related to chemical properties. Six items each comprised the facets for association of risk with routes of entry into the body and association of risk with adverse health outcomes of pesticide exposure. The items appeared in random order, as determined by a random number generator, rather than according to facet. Six items were reverse coded. For the purpose of scoring and analyzing the *Pesticide Risk Beliefs Inventory* data, the response “strongly disagree” corresponded to a score of 1, “disagree” corresponded to 2, “somewhat disagree” corresponded to 3, “somewhat agree” corresponded to 4, “agree” corresponded to 5, and “strongly agree” corresponded to 6. For reverse-coded items, “strongly disagree” corresponded to a numerical score of 6 and so forth. Each of the four items related to determination of risk using physical properties were scored as reverse-coded items so that a higher score relates to a more expert-like belief. For all inventory items, a score of 4 or higher represents agreement with expert beliefs.

Six experts in the field of science education reviewed the items for reading level. This review resulted in re-writing one item. The item originally included language that might not be accessible for the general public: “I can determine if a pesticide is dangerous by knowing its *chemical structure*.” To address chemical structure in a way that would not draw as heavily on scientific terminology, the item was replaced with “I can determine if a pesticide is dangerous by knowing the family of chemicals that the pesticide belongs to”. Using the Flesch-Kincaid formula for assessing readability, the grade level scores for the inventory items ranged from 6.94 to 12.31. Expert review of the item relevance and content coverage provided evidence of content validity for this inventory [33].

In addition to assisting in item development, experts in pesticide toxicology evaluated the finished product. One revision based on expert review of the completed inventory included the deletion of the following item: “I can determine if a pesticide is dangerous by knowing whether it kills bugs, weeds, or plant diseases.” Although the classes of pesticides associated with the most symptomatic illnesses are insecticides (*i.e.*, organophosphates, pyrethrins, and pyrethroids) [34], an entomologist described the concern that a range of toxicity levels exists within the broad category of insecticides: “An insecticide can range from everything from insecticidal soap to Temik^®^ (a toxic carbamate), so just knowing if it’s an insecticide, herbicide or fungicide doesn’t tell you anything at all about how it will affect people or what environmental effects it may have” [35]. Removing this question improved facet and inventory internal consistency.

### 2.2. Administering the Inventory: Testing and Validation

A sample of 43 farmworker pesticide educators from one state in the southeastern United States participated in the testing of the *Pesticide Risk Beliefs Inventory*. Seventeen pesticide educators who attended one of two pesticide education workshops provided by a land-grant university in the southeastern U.S. completed pen-and-paper versions of the inventory. The link to an online version of the inventory was distributed using relevant professional electronic subscription lists (*i.e.*, listservs). Twenty-six online respondents completed the online questionnaire. Responses were scored, facet means and Cronbach’s alpha values were determined, and the inventory Cronbach’s alpha was calculated. For survey submissions missing fewer than 15% of item responses (*i.e.*, 3), the respondent’s average score for the missing item’s corresponding facet was used as the response.

The sample was predominantly female (n = 26) and comprised largely of White/European American (n = 15) and Latino/Hispanic individuals (n = 24). Education levels ranged from the high school diploma to the doctoral degree. Various employer organizations represented in the sample included migrant and community health centers, Cooperative Extension, state agencies, Migrant Education, Migrant Head Start, and farmworker advocacy groups. These pesticide educators reported that they typically provide as few as one and as many as 600 pesticide lessons to farmworkers each year.

## 3. Results and Discussion

### 3.1. Results

#### 3.1.1. Reliability and Validity

Cronbach’s alpha internal consistency estimate is a reliability measurement that is sensitive to content sampling error and that assesses the content homogeneity of the inventory [33]. Findings from this study indicated an adequate internal consistency when the inventory was used with the pesticide educator study group. Using the sample of 43 pesticide educators, the Cronbach’s alpha for the entire inventory was found to be 0.780. The Cronbach’s alpha values for the individual facets ranged from 0.758 to 0.864 (Table 2). Expert review of the item relevance and content coverage provided evidence of content validity for this inventory.

#### 3.1.2. Pesticide Educators’ Beliefs

Pesticide educators’ composite scores on the inventory ranged from 64 to 111. The mean score was 90.8. In all four facets, the sample was in agreement with expert-like beliefs regarding pesticide risk. For all but two items, which received ambivalent average responses of 3.442 (related to concern about losing the ability to have children with pesticide exposure) and 3.558 (related to concern about visiting the emergency room due to pesticide exposure), all items were answered with general agreement, indicating alignment with expert-like beliefs.

Pesticide educators strongly disagreed with the belief that they could use physical properties of a pesticide to determine risk. They most strongly disagreed with using the taste (5.581) and color (5.488), respectively, to assess the danger posed by a pesticide. In strongly disagreeing with the belief that pesticide risk can be determined by a pesticide’s physical properties, the sample of pesticide educators exhibited agreement with expert-like beliefs. The sample less strongly agreed that they could use chemical properties of pesticides to determine risk. The lowest average scores were for the items related to using the ingredients (4.209) and the chemical family (4.233) to determine risk.

The sample agreed that pesticide risk was associated with the routes of entry of the pesticides into the body (4.988) and with the adverse health effects (4.271). The sample indicated the greatest concern with the skin as a route of entry (5.395). They agreed that a poisoning event (4.842) was a concern but were less worried about losing the ability to have children (3.442) and having to visit the emergency room (3.558).

### 3.2. Discussion

In this study, the *Pesticide Risk Beliefs Inventory* was developed and tested with a group of pesticide educators as a first attempt to quantitatively assess mental models of pesticide risks. Disparate studies have revealed misconceptions and knowledge gaps in lay conceptualizations of pesticide risks, and these findings informed the selection of inventory items. Expert beliefs about pesticide risks were derived from the literature, confirmed by pesticide toxicologists, and likewise used to create inventory items regarding important beliefs. Testing of the inventory demonstrated the psychometric strength of this instrument, using the standard that a reliability estimate of 0.7 or higher is appropriate [36], with the inventory Cronbach’s alpha of 0.780. Expert review supported content validity. The extent to which findings regarding educators’ beliefs are as expected and the internal reliability estimates are adequate provide weak evidence for the instrument’s construct validity; future work might focus on more rigorous testing of construct validity. Morgan *et al.* [9] proposed the usefulness of confirmatory questionnaires, like the inventory used in this study, for assessing the prior knowledge of the audience, focusing the content of risk communications (e.g., chemical properties useful in assessing pesticide toxicity), and evaluating the effectiveness of risk communication.

In general, the pesticide risk beliefs of pesticide educators in this southeastern state aligned with an expert perspective. This finding is reassuring because pesticide educators have the critical task of communicating risks to the special risk farmworker population. Despite the pesticide educators’ having generally high (expert-like) scores on the inventory, the inventory was useful in identifying possible areas for professional development. Although the sample strongly disagreed with the belief that physical properties of a pesticide might be useful in determining risk, they less strongly agreed in the usefulness of chemical properties. While pesticide educators rejected the lay belief, they were less committed to the expert belief. This finding suggests that professional development programs for pesticide educators might focus on discussions of chemical families and ingredients. The survey could be re-administered following the professional development program to assess the presence of more expert-like beliefs.

Given that highly technical questions regarding pesticides and their risks were not asked, an assessment of pesticide educators’ knowledge and beliefs of technical information—beyond basic beliefs related to exposure, routes of entry, and health outcomes—cannot be made. The importance of beliefs in shaping practices [13], however, suggests the centrality of identifying pesticide educator basic beliefs about the content of their risk communications in understanding their teaching and communication practices.

This study was designed to assess risk beliefs of pesticide educators in a southeastern state. As such, the items and language used were determined by a group of experts in both pesticide toxicology and science education and validated with pesticide educators ranging from those with a high school education to the doctoral degree and including both native Spanish and English speakers. A possible limitation of this study is that the instrument, its items, and its scaling have not yet been validated with migrant farmworkers or with individuals representing a full range of ethnicities (beyond Latino and European American) and having experiences with pesticides that would be reflective of the general public. Researchers would be advised to validate this instrument with groups that differ greatly from the educators in this study.

Given the results of this study, a recommended next step is administering the inventory to larger samples and different groups, especially farmworkers (including those in other countries) and the general public, who may demonstrate more of the lay beliefs regarding pesticide risk documented in the literature. The low literacy levels and limited formal education of the farmworker population [6,37], may require oral administration of the inventory for all non-readers.

## 4. Conclusions

The *Pesticide Risk Beliefs Inventory* developed in this study has been shown to be psychometrically sound. Our testing demonstrated its ability to gauge pesticide beliefs in a group of pesticide educators in a southeastern state, who express predominantly expert beliefs. This inventory holds promise for exploring beliefs about pesticide risks for a variety of other audiences—students, secondary science teachers, college science professors, the agricultural labor force, and the general public. The inventory could be used to compare beliefs among different groups or to prepare relevant risk communication materials, professional development sessions, and informal science lessons. By using this inventory, scientists and science educators can pre-assess beliefs and content knowledge as well as post-assess the effectiveness of risk communications in addressing misconceptions, filling knowledge gaps, and expanding expert-like beliefs.

## Figures and Tables

**Table 1.** Pesticide risk facets and items used in the *Pesticide Risk Beliefs Inventory*.

**Item No.**	**Facet**	**Item**
1	Physical Properties	I can determine if a pesticide is dangerous by its smell.
2	Routes of Entry	When I am working with a pesticide, I am worried about having the pesticide enter my body when I breathe.
3	Chemical Properties	I can determine if a pesticide is dangerous by reading its chemical label.
4	Adverse Health Outcomes	When I am working with a pesticide, I am not worried about getting cancer in the future.
5	Physical Properties	I can determine if a pesticide is dangerous by seeing whether it is a powder, liquid, or granule.
6	Adverse Health Outcomes	When I am working with a pesticide, I am worried about having a recurrent problem with my skin.
7	Routes of Entry	When I am working with a pesticide, I am not concerned about covering my nose.
8	Routes of Entry	When I am working with a pesticide, I am not worried about having the pesticide enter my body through my skin.
9	Adverse Health Outcomes	When I am working with a pesticide, I am worried about having to go to the emergency room.
10	Physical Properties	I can determine if a pesticide is dangerous by its color.
11	Routes of Entry	When I am working with a pesticide, I am concerned about covering my skin.
12	Chemical Properties	I can determine if a pesticide is dangerous by knowing the family of chemicals that the pesticide belongs to.
13	Chemical Properties	I can determine if a pesticide is dangerous by knowing its ingredients.
14	Routes of Entry	When I am working with a pesticide, I am not worried about having the pesticide enter my body through my eyes.
15	Adverse Health Outcomes	When I am working with a pesticide, I am worried about losing my ability to have children.
16	Routes of Entry	When I am working with a pesticide, I am worried about having the pesticide enter my body when I eat or drink.
17	Adverse Health Outcomes	When I am working with a pesticide, I am not worried about having difficulty breathing.
18	Adverse Health Outcomes	When I am working with a pesticide, I am not worried about being poisoned.
19	Physical Properties	I can determine if a pesticide is dangerous by its taste.

**Table 2.** *Pesticide Risk Beliefs Inventory* Facets with Cronbach’s Alpha and Mean Values.

**Facet**	**Facet Cronbach’s Alpha**	**Facet Mean**
Determination of Risk Using Physical Properties	0.864	5.390
Determination of Risk Using Chemical Properties	0.805	4.574
Risk Associated with Routes of Entry into Body	0.758	4.988
Risk Associated with Adverse Health Effects	0.782	4.271
